# Supplementary material for: Genome-wide identification of the trehalose-6-phosphate synthase gene family in sweet orange (Citrus sinensis) and expression analysis in response to phytohormones and abiotic stresses
Source: PeerJ. 2022 Sep 9;10:e13934. doi: 10.7717/peerj.13934 (PMC9466596; doi:10.7717/peerj.13934)
Supplement: Supplemental Information 3 [file peerj-10-13934-s003.docx]

| **Gene name** | **Primer name** | **Sequence (5′–3′)** |
| --- | --- | --- |
| *FBOX* | boxF | TTGGAAACTCTTTCGCCACT |
|  | boxR | CAGCAACAAAATACCCGTCT |
| *CisTPS1* | 1F | TTAGGAGGGGTGAGGACTCG |
|  | 1R | CCAGCCCAACCAATCCATCT |
| *CisTPS2* | 2F | GACGTTGTTGGGGAATTGGC |
|  | 2R | TGGGGCATGACAGTTCCATC |
| *CisTPS3* | 3F | CACGGCATTTCTTGTCCTGC |
|  | 3R | AAACCTTTGCCTCCGTTCCA |
| *CisTPS4* | 4F | TGGGGCCATTCGAGTAAACC |
|  | 4R | GCAAAAAGCTACGAGCCCAG |
| *CisTPS5* | 5F | GATTGTTGCTTTGGGGCCTG |
|  | 5R | GTGGCATCACAGTCCCATCA |
| *CisTPS6* | 6F | GGCATCAGTGTGTCCACGTA |
|  | 6R | TGCATCAGCTACGGCATCAA |
| *CisTPS7* | 7F | ACATTTGCTGGTCGGAAGGT |
|  | 7R | GCAAAACAACTTTGCCACGC |
| *CisTPS8* | 8F | TCTCCTCGGACACTGAGGTT |
|  | 8R | GGTAGAAAGGTCGGCACACA |
