# Supplementary material for: Genome-wide identification of the trehalose-6-phosphate synthase gene family in sweet orange (Citrus sinensis) and expression analysis in response to phytohormones and abiotic stresses
Source: PeerJ. 2022 Sep 9;10:e13934. doi: 10.7717/peerj.13934 (PMC9466596; doi:10.7717/peerj.13934)
Supplement: Supplemental Information 4 [file peerj-10-13934-s004.docx]

| **Protein** | **Alpha helix (%)** | **Beta turn (%)** | **Random coil (%)** | **Extended strand (%)** |
| --- | --- | --- | --- | --- |
| CisTPS1 | 42.68 | 6.05 | 36.94 | 14.33 |
| CisTPS2 | 42.21 | 4.67 | 36.33 | 16.59 |
| CisTPS3 | 45.01 | 5.05 | 32.85 | 17.09 |
| CisTPS4 | 42.53 | 4.87 | 36.04 | 16.57 |
| CisTPS5 | 42.39 | 5.39 | 35.25 | 16.98 |
| CisTPS6 | 42.67 | 4.93 | 35.10 | 17.31 |
| CisTPS7 | 43.44 | 5.12 | 37.78 | 13.66 |
| CisTPS8 | 43.09 | 4.88 | 34.61 | 17.42 |
