# Supplementary material for: Genome-wide identification of the trehalose-6-phosphate synthase gene family in sweet orange (Citrus sinensis) and expression analysis in response to phytohormones and abiotic stresses
Source: PeerJ. 2022 Sep 9;10:e13934. doi: 10.7717/peerj.13934 (PMC9466596; doi:10.7717/peerj.13934)
Supplement: Supplemental Information 5 [file peerj-10-13934-s005.docx]

| **Seq_1** | **Seq_2** | **Ka** | **Ks** | **Ka/Ks** | **EffectiveLen** | **AverageS-sites** | **AverageN-sites** | **cN** | **cS** | **pN** | **pS** |
| --- | --- | --- | --- | --- | --- | --- | --- | --- | --- | --- | --- |
| XM_006467546.3 | AT1G06410.1 | 0.1565 | 3.4731 | 0.0451 | 2550 | 570 | 1980 | 279.6667 | 423.3333 | 0.1412 | 0.7427 |
| XM_006474056.3 | AT2G18700.1 | 0.2259 | NaN | NaN | 2514 | 550.75 | 1963.25 | 383 | 422 | 0.1950 | 0.7662 |
| XM_006476690.3 | AT1G23870.1 | 0.2030 | 3.2907 | 0.0617 | 2532 | 578.75 | 1953.25 | 347.3333 | 428.6667 | 0.1778 | 0.7407 |
| XM_006476690.3 | AT1G60140.5 | 0.2056 | 2.0463 | 0.1005 | 2532 | 586.4166667 | 1945.5833 | 349.9167 | 411.0833 | 0.1799 | 0.7010 |
| XM_006476690.3 | AT1G70290.1 | 0.2157 | 3.2207 | 0.0670 | 2517 | 576.0833333 | 1940.9166 | 363.8333 | 426.1667 | 0.1875 | 0.7398 |
| XM_006477757.3 | AT1G78580.1 | 0.1419 | 1.3393 | 0.1060 | 2778 | 654.25 | 2123.75 | 274.5833 | 408.4167 | 0.1293 | 0.6243 |
| XM_006477757.3 | AT1G16980.1 | 0.2317 | 1.4380 | 0.1611 | 2349 | 546.8333333 | 1802.1666 | 359.1667 | 349.8333 | 0.1993 | 0.6397 |
| XM_006483756.3 | AT1G23870.1 | 0.1339 | 2.4904 | 0.0538 | 2580 | 587.3333333 | 1992.6666 | 244.4167 | 424.5833 | 0.1227 | 0.7229 |
| XM_006483756.3 | AT1G60140.5 | 0.1401 | 1.6036 | 0.0874 | 2583 | 597.4166667 | 1985.5833 | 253.75 | 395.25 | 0.1278 | 0.6616 |
| XM_006483756.3 | AT1G70290.1 | 0.1482 | 2.6017 | 0.0570 | 2556 | 583.1666667 | 1972.8333 | 265.25 | 423.75 | 0.1345 | 0.7266 |
