# Supplementary material for: Genome-wide identification of the trehalose-6-phosphate synthase gene family in sweet orange (Citrus sinensis) and expression analysis in response to phytohormones and abiotic stresses
Source: PeerJ. 2022 Sep 9;10:e13934. doi: 10.7717/peerj.13934 (PMC9466596; doi:10.7717/peerj.13934)
Supplement: Supplemental Information 6 [file peerj-10-13934-s006.docx]

| **Motif ID** | **Conserved Motifs** | **E-value** | **Width** | **Sites** |
| --- | --- | --- | --- | --- |
| Motif 1 | FFLHSPFPSSEIYRTLPVRDEJLRALLNADLIGFHTFDYARHFLSCCSRM | 1.3e-247 | 50 | 8 |
| Motif 2 | WKQIAEPVMKLYTETTDGSYIEDKETALVWHYQDADPDFGSCQAKELLDH | 1.1e-191 | 50 | 8 |
| Motif 3 | LSGAIRVNPWNIDAVADAMDSAJNMSDAEKQLRHEKHYRYVSTHDVAYWA | 5.1e-190 | 50 | 8 |
| Motif 4 | LGVDDMDIFKGISLKLLAMEQLLEQHPEWRGKVVLVQIANPARGRGKDVQ | 2.8e-172 | 50 | 8 |
| Motif 5 | KRINETFGKPGYEPVVLIDRPLDFYEKIAYYAIAECCLVTAVRDGMNLVP | 3.1e-139 | 50 | 8 |
| Motif 6 | VMEVINPEDDYVWVHDYHLMVLPTFLRKRFNRVKLG | 2.5e-138 | 36 | 8 |
| Motif 7 | SQKLLDEFKCVPTFLPPDLVKKYYHGFCKQHLWPLFHYMLP | 4.6e-156 | 41 | 8 |
| Motif 8 | PDFVLCIGDDRSDEDMFESIKSAVAGPSLPPVAEVFACTVGQKPSKAKYY | 1.9e-134 | 50 | 6 |
| Motif 9 | KRGYIGLEYFGRTVSIKILPVGIHMGRLESVLNLPETZAKVKELKEQFKG | 2.1e-122 | 50 | 8 |
| Motif 10 | RSFLQDLERACRDHYRKRCWGIGLGLGFRVVALDPNFRKLSIDHIVSAY | 4.7e-109 | 49 | 6 |
| Motif 11 | LESVLANEPVVVKRGQNIVEVKPQGVSKG | 1.3e-082 | 29 | 8 |
| Motif 12 | EWFSPCENLGJAAEHGYFLRW | 3.6e-059 | 21 | 8 |
| Motif 13 | GGRFDRSLWQAYVSANKIFAD | 2.4e-058 | 21 | 8 |
| Motif 14 | KPSPEVISILNTLCNDPKNTVFIVSGRGR | 2.2e-043 | 29 | 8 |
| Motif 15 | SGGWCFSWDEDSLLLQLKDGLPEDTEVIY | 1.5e-045 | 29 | 6 |
| Motif 16 | KSMLVVSEFIGCSPS | 1.5e-038 | 15 | 8 |
| Motif 17 | PSSVSRERLIIVANQLPVKAKRRSE | 1.3e-031 | 25 | 8 |
| Motif 18 | RTNNRAILLDYDGTVMPPTSI | 4.6e-025 | 21 | 7 |
| Motif 19 | RRLPRVMTVPGVISELDDDESN | 2.5e-020 | 22 | 6 |
| Motif 20 | MVSRSYSNLLDLASGDFPDFP | 1.1e-014 | 21 | 5 |
