# Supplementary material for: Genome-wide identification of the trehalose-6-phosphate synthase gene family in sweet orange (Citrus sinensis) and expression analysis in response to phytohormones and abiotic stresses
Source: PeerJ. 2022 Sep 9;10:e13934. doi: 10.7717/peerj.13934 (PMC9466596; doi:10.7717/peerj.13934)
Supplement: Supplemental Information 7 [file peerj-10-13934-s007.docx]

| **No.** | **Site name** | **Function** | **Gene** |
| --- | --- | --- | --- |
|  | 3-AF1 binding site | light response | *CisTPS2, CisTPS7* |
|  | AACA_motif | Endosperm expression | *CisTPS8* |
|  | AAGAA-motif |  | *CisTPS3, CisTPS4, CisTPS6* |
|  | A-box | cis-acting regulatory element | *CisTPS2* |
|  | ABRE | abscisic acid response | *CisTPS1, CisTPS2, CisTPS3, CisTPS5, CisTPS6, CisTPS7, CisTPS8* |
|  | ABRE2 |  | *CisTPS2, CisTPS3,* |
|  | ABRE3a |  | *CisTPS1, CisTPS2, CisTPS3, CisTPS8* |
|  | ABRE4 |  | *CisTPS1, CisTPS2, CisTPS3, CisTPS8* |
|  | ACE | light response | *CisTPS2, CisTPS3, CisTPS8* |
|  | AC-II |  | *CisTPS6* |
|  | AE-box | light response | *CisTPS3, CisTPS8* |
|  | AP-1 |  | *CisTPS3, CisTPS4, CisTPS6* |
|  | ARE | oxygen response | *CisTPS1, CisTPS2, CisTPS3, CisTPS4, CisTPS5, CisTPS8* |
|  | as-1 |  | *CisTPS1, CisTPS2, CisTPS3, CisTPS4, CisTPS6, CisTPS7, CisTPS8* |
|  | AT~ABRE |  | *CisTPS2* |
|  | AT~TATA-box |  | *CisTPS1, CisTPS3, CisTPS4, CisTPS5, CisTPS6, CisTPS7, CisTPS8* |
|  | AT1-motif | light response | *CisTPS3, CisTPS7* |
|  | ATC-motif | light response | *CisTPS3* |
|  | ATCT-motif | light response | *CisTPS3, CisTPS7* |
|  | AT-rich element | binding site of ATBP-1 | *CisTPS3* |
|  | AuxRR-core | auxin respons | *CisTPS2,* |
|  | Box 4 | light response | *CisTPS1, CisTPS2, CisTPS3, CisTPS4, CisTPS5, CisTPS6, CisTPS7, CisTPS8* |
|  | Box II | light response | *CisTPS2, CisTPS3,* |
|  | CAAT-box | common cis-acting element | *CisTPS1, CisTPS2, CisTPS3, CisTPS4, CisTPS5, CisTPS6, CisTPS7, CisTPS8* |
|  | CARE |  | *CisTPS1, CisTPS5,* |
|  | CAT-box | meristem expression | *CisTPS1, CisTPS4* |
|  | CCAAT-box | MYBHv1 binding site | *CisTPS5, CisTPS7* |
|  | CCGTCC motif |  | *CisTPS2* |
|  | CGTCA-motif | MeJA-response | *CisTPS1, CisTPS2, CisTPS3, CisTPS4, CisTPS6, CisTPS7, CisTPS8* |
|  | CTAG-motif |  | *CisTPS1* |
|  | DRE1 |  | *CisTPS5, CisTPS8* |
|  | ERE |  | *CisTPS1, CisTPS2, CisTPS3, CisTPS4, CisTPS5, CisTPS6, CisTPS7, CisTPS8* |
|  | Gap-box | light response | *CisTPS1* |
|  | GARE-motif | gibberellin response | *CisTPS1* |
|  | GATA-motif | light response | *CisTPS4, CisTPS5* |
|  | GATT-motif | light response | *CisTPS6* |
|  | G-box | light response | *CisTPS1, CisTPS2, CisTPS3, CisTPS5, CisTPS6, CisTPS7, CisTPS8* |
|  | GC-motif | oxygen response | *CisTPS6* |
|  | GCN4_motif | endosperm expression | *CisTPS1, CisTPS3, CisTPS4, CisTPS6* |
|  | GT1-motif | light response | *CisTPS1, CisTPS2, CisTPS3, CisTPS6, CisTPS8* |
|  | HD-Zip 1 | differentiation of the palisade mesophyll cells | *CisTPS3* |
|  | I-box | light response | *CisTPS1, CisTPS2, CisTPS5* |
|  | LAMP-element | light response | *CisTPS5, CisTPS6, CisTPS8* |
|  | LTR | low-temperature response | *CisTPS1, CisTPS3, CisTPS6* |
|  | MBS | drought respons | *CisTPS2, CisTPS5, CisTPS6, CisTPS7* |
|  | MRE | light response | *CisTPS1, CisTPS3, CisTPS4,* |
|  | MYB |  | *CisTPS1, CisTPS2, CisTPS3, CisTPS4, CisTPS5, CisTPS6, CisTPS7, CisTPS8* |
|  | Myb |  | *CisTPS2, CisTPS3, CisTPS4, CisTPS5, CisTPS6, CisTPS7, CisTPS8* |
|  | MYB recognition site |  | *CisTPS5, CisTPS7* |
|  | Myb-binding site |  | *CisTPS1, CisTPS3, CisTPS4, CisTPS8* |
|  | MYB-like sequence |  | *CisTPS1, CisTPS2, CisTPS4, CisTPS5, CisTPS6, CisTPS8* |
|  | MYC |  | *CisTPS1, CisTPS2, CisTPS3, CisTPS4, CisTPS5, CisTPS6, CisTPS7, CisTPS8* |
|  | Myc |  | *CisTPS5, CisTPS6, CisTPS7, CisTPS8* |
|  | O2-site | zein metabolism | *CisTPS2, CisTPS8* |
|  | P-box | gibberellin response | *CisTPS8* |
|  | Sp1 | light response | *CisTPS1,* |
|  | STRE |  | *CisTPS1, CisTPS2, CisTPS4, CisTPS5, CisTPS8* |
|  | TATA |  | *CisTPS1, CisTPS3, CisTPS4, CisTPS5, CisTPS8* |
|  | TATA-box | core promoter element | *CisTPS1, CisTPS2, CisTPS3, CisTPS4, CisTPS5, CisTPS6, CisTPS7, CisTPS8* |
|  | TATC-box | gibberellin response | *CisTPS4, CisTPS5* |
|  | TCA |  | *CisTPS4, CisTPS6, CisTPS7, CisTPS8* |
|  | TCA-element | salicylic acid response | *CisTPS1, CisTPS4, CisTPS6* |
|  | TCCC-motif | light response | *CisTPS2, CisTPS4* |
|  | TC-rich repeats | defense and stress response | *CisTPS1* |
|  | TCT-motif | light response | *CisTPS1, CisTPS2, CisTPS3, CisTPS4, CisTPS7, CisTPS8* |
|  | TGACG-motif | MeJA response | *CisTPS1, CisTPS2, CisTPS3, CisTPS4, CisTPS6, CisTPS7, CisTPS8* |
|  | TGA-element | auxin respons | *CisTPS1, CisTPS3, CisTPS5, CisTPS7* |
|  | W box |  | *CisTPS1, CisTPS4, CisTPS6, CisTPS7, CisTPS8* |
|  | WRE3 |  | *CisTPS2, CisTPS3,* |
|  | WUN-motif | Wound response | *CisTPS3, CisTPS4, CisTPS5, CisTPS6, CisTPS7, CisTPS8* |
